# Supplementary figures and images for: Large scale meta-analysis of preclinical toxicity data for target characterisation and hypotheses generation
Source: PLoS One. 2021 Jun 8;16(6):e0252533. doi: 10.1371/journal.pone.0252533 (PMC8186779; doi:10.1371/journal.pone.0252533)

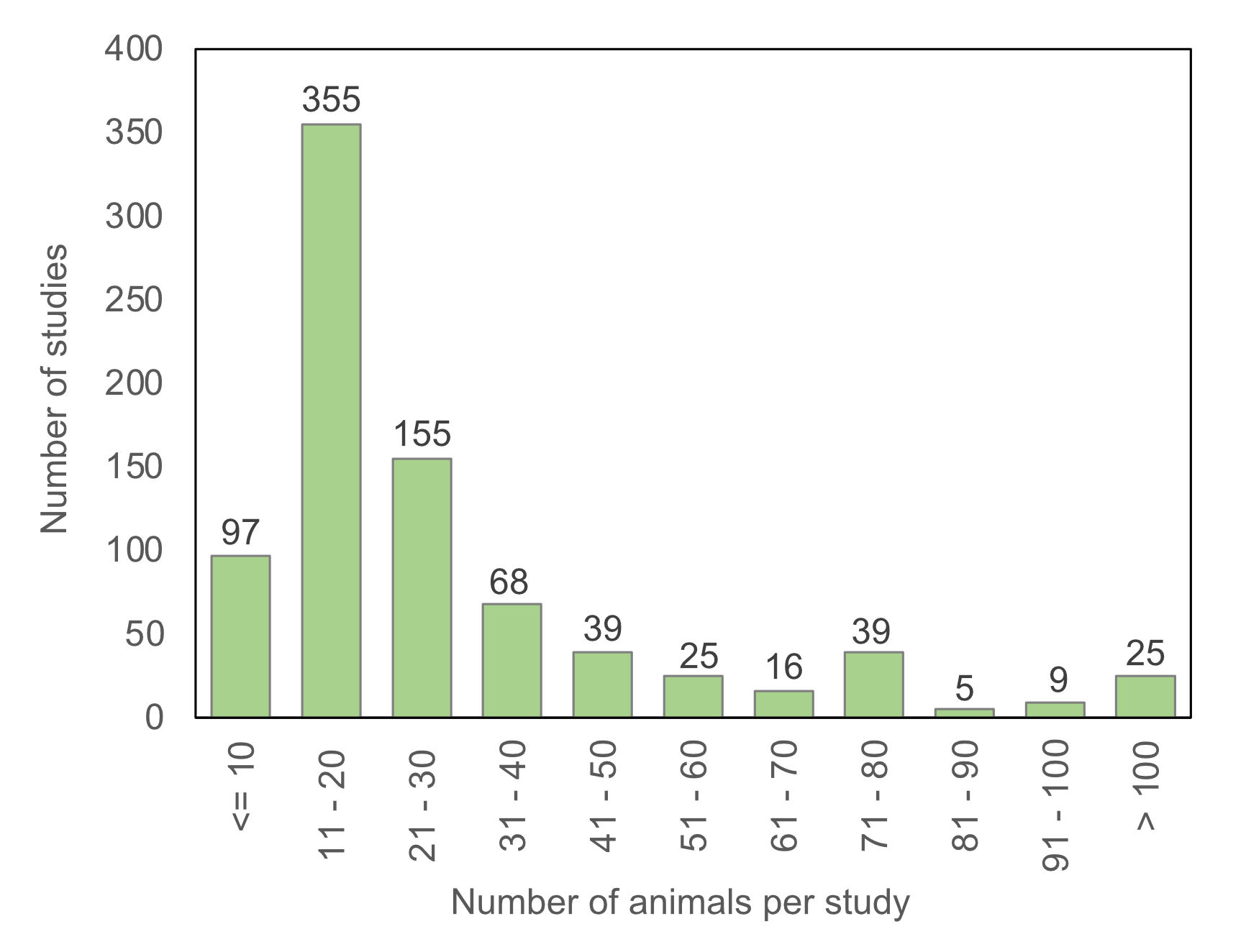

Supplement: S1 Fig — (TIF) [file pone.0252533.s005.tif]

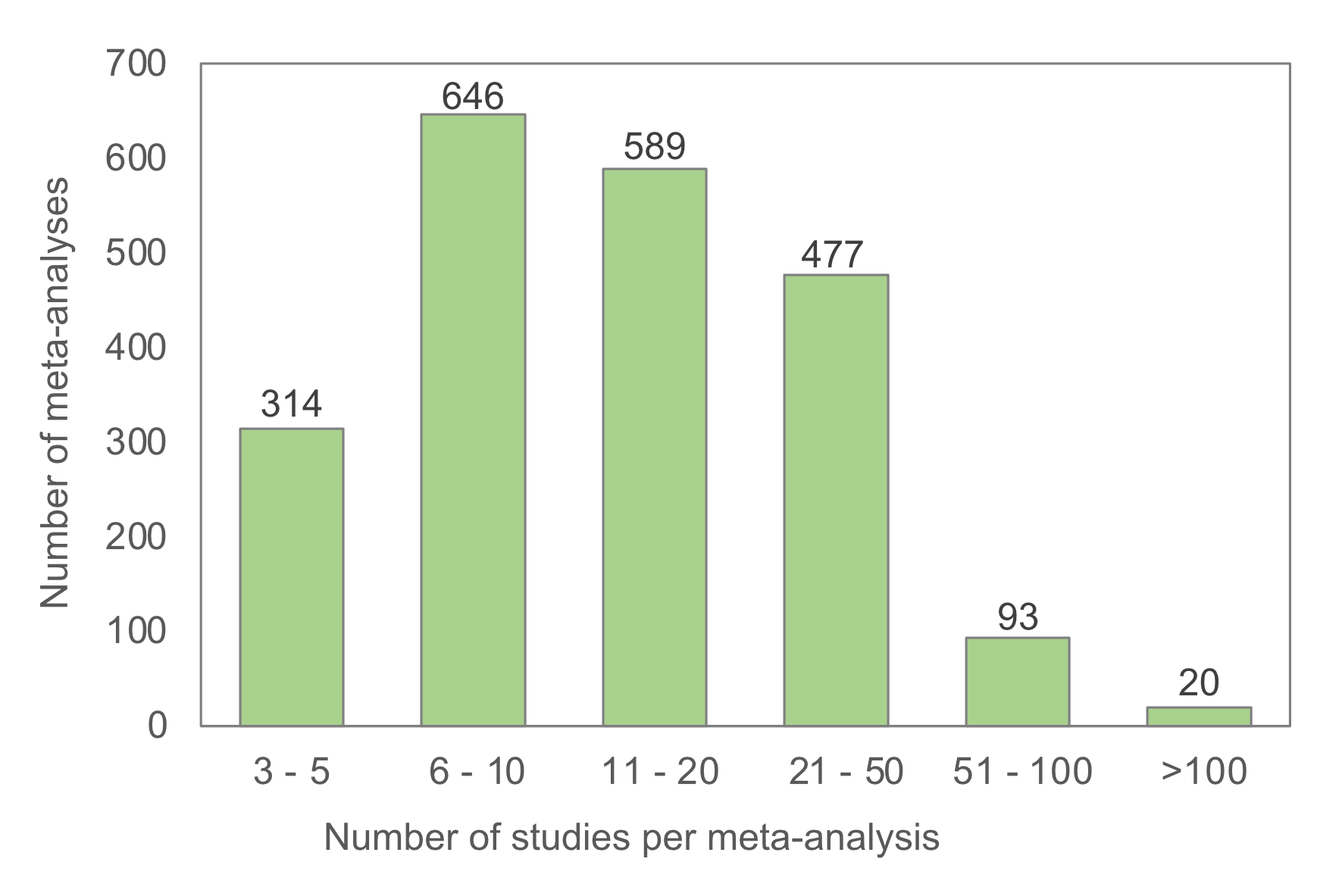

Supplement: S2 Fig — (TIF) [file pone.0252533.s006.tif]

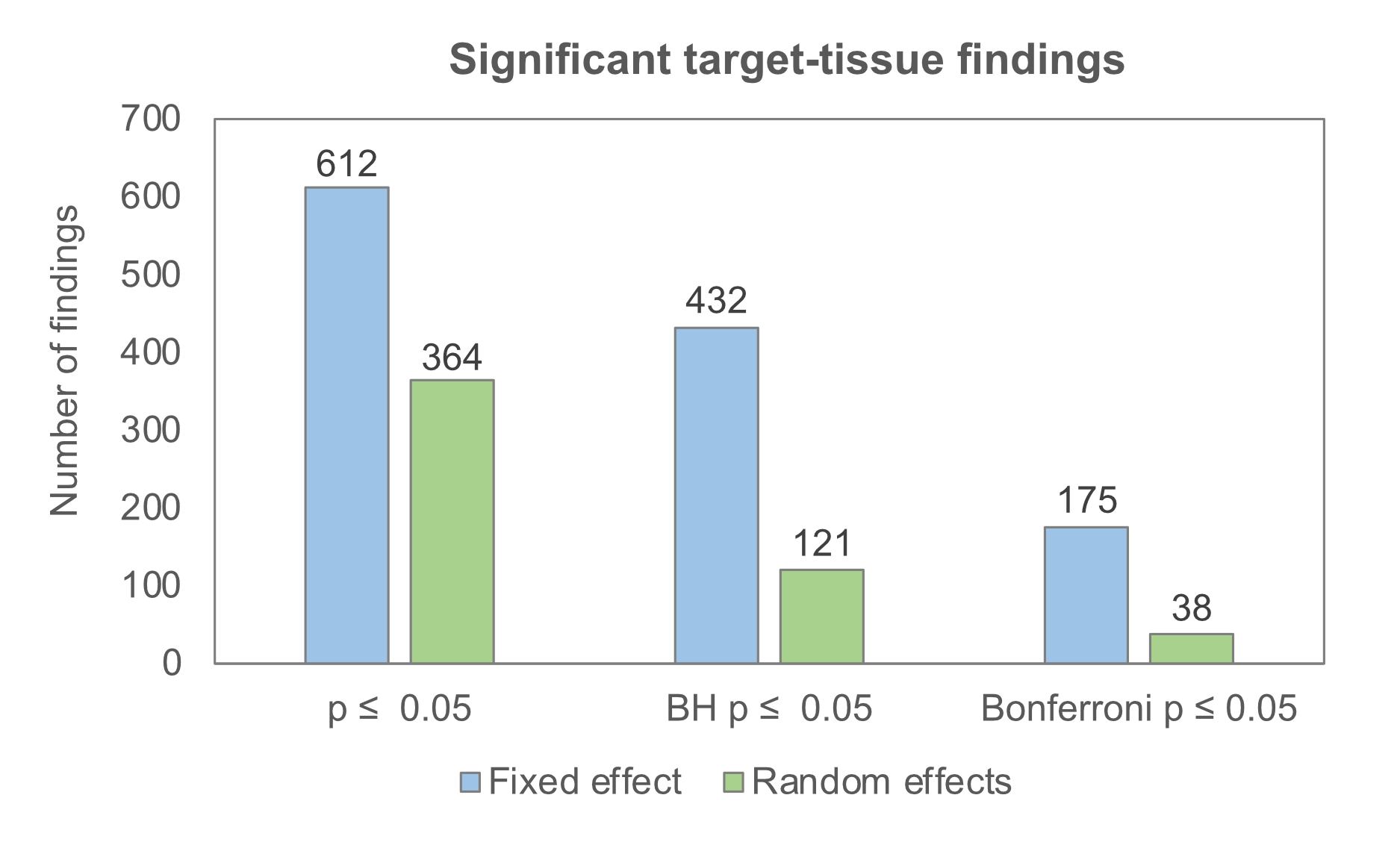

Supplement: S3 Fig — (TIF) [file pone.0252533.s007.tif]

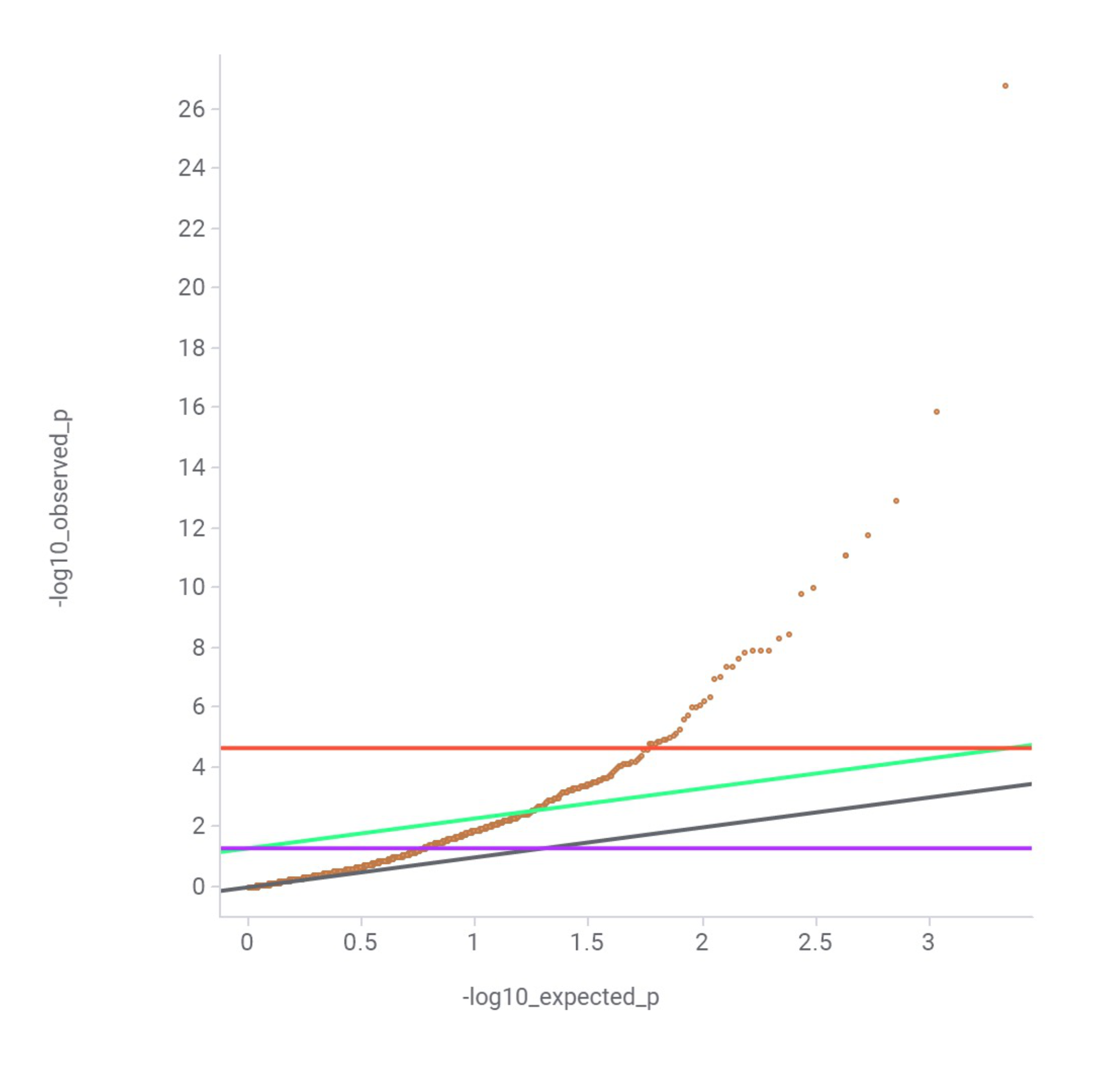

Supplement: S4 Fig — Line representing unadjusted p value cut-off (0.05) in magenta. Line representing Benjamini-Hockberg 5% false discovery rate (FDR) in green. Line representing Bonferroni adjustment in red. The plot compares the astringencies of the different methods (points above the lines would be considered significant associations after adjustment). (TIF) [file pone.0252533.s008.tif]

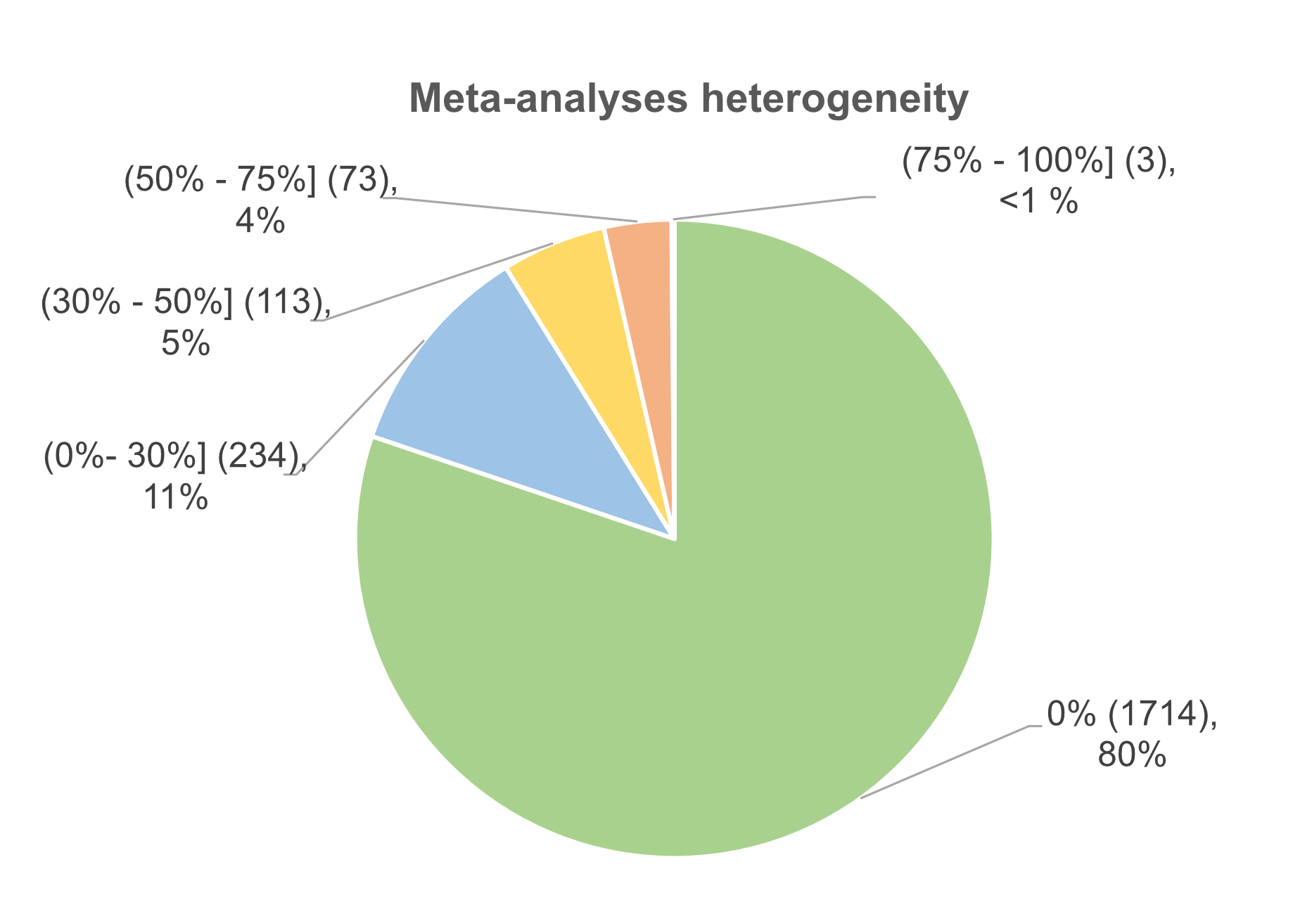

Supplement: S5 Fig — (TIF) [file pone.0252533.s009.tif]

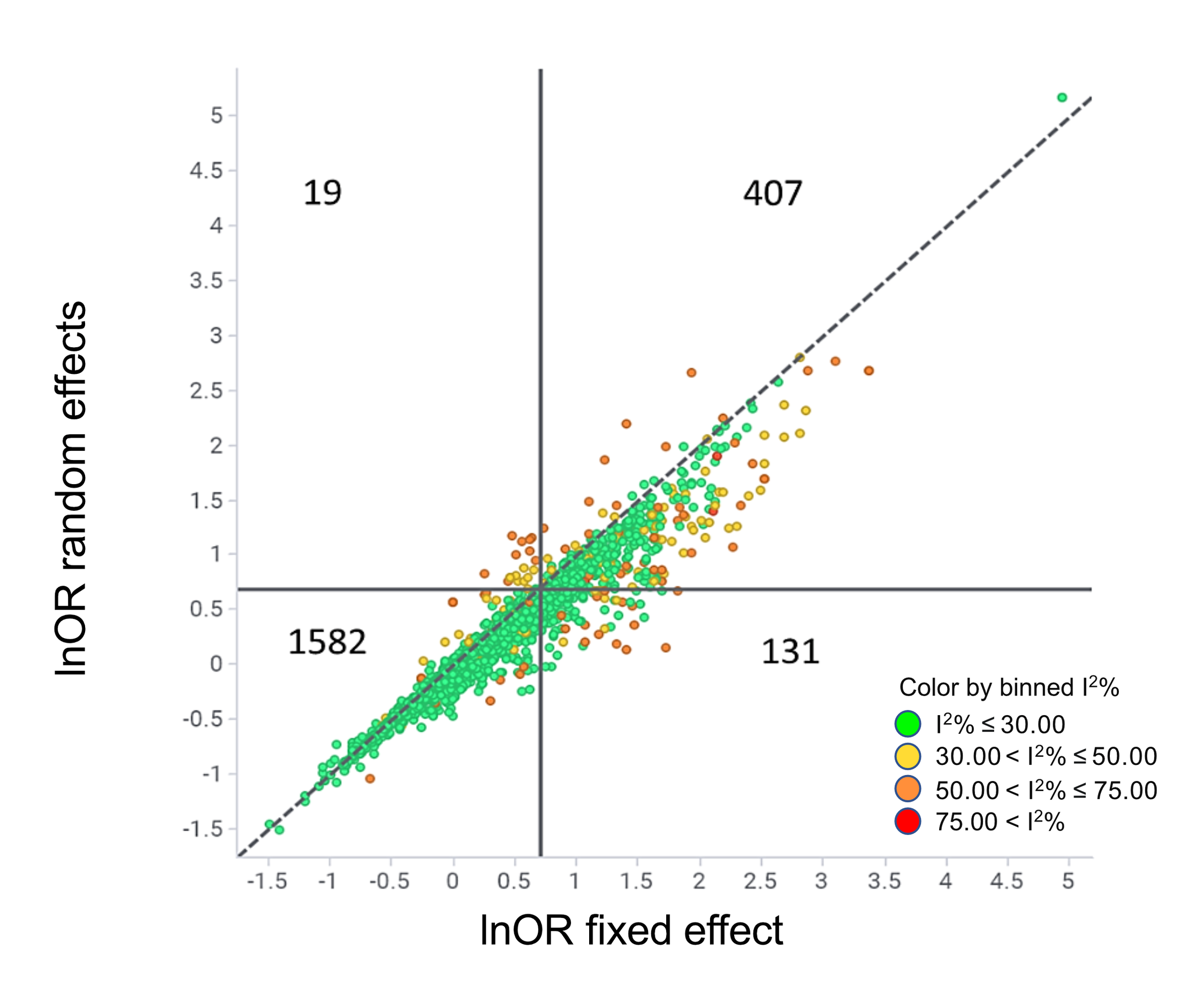

Supplement: S6 Fig — Numbers in the quadrants separated by the horizontal and vertical lines represent the number of meta-analyses for which the aggregated effect size falls within the quadrant. Datapoints are coloured by the heterogeneity (I2) in the set of studies included in the meta-analysis represented by the datapoint. (TIF) [file pone.0252533.s010.tif]

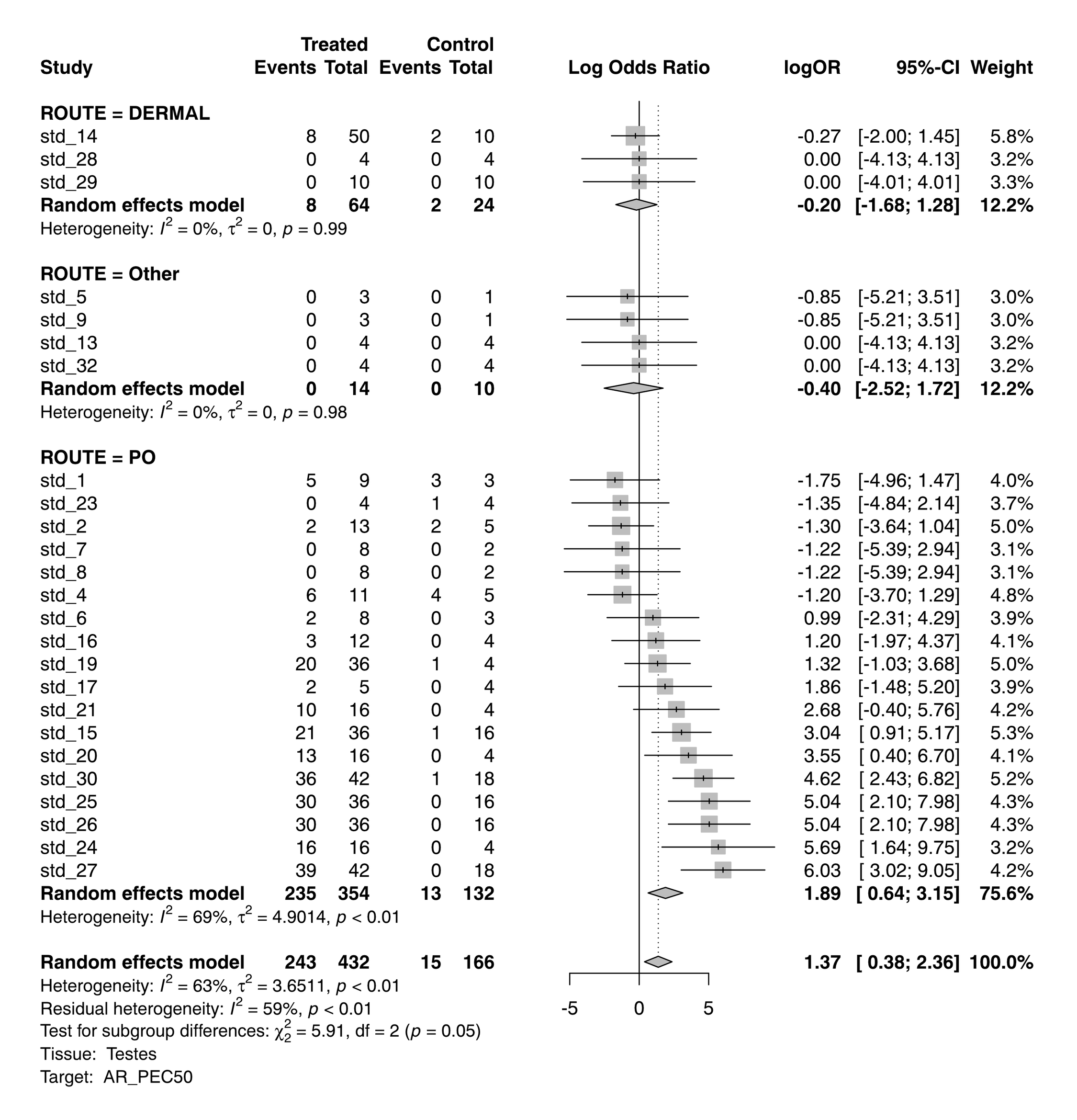

Supplement: S7 Fig — (TIF) [file pone.0252533.s011.tif]

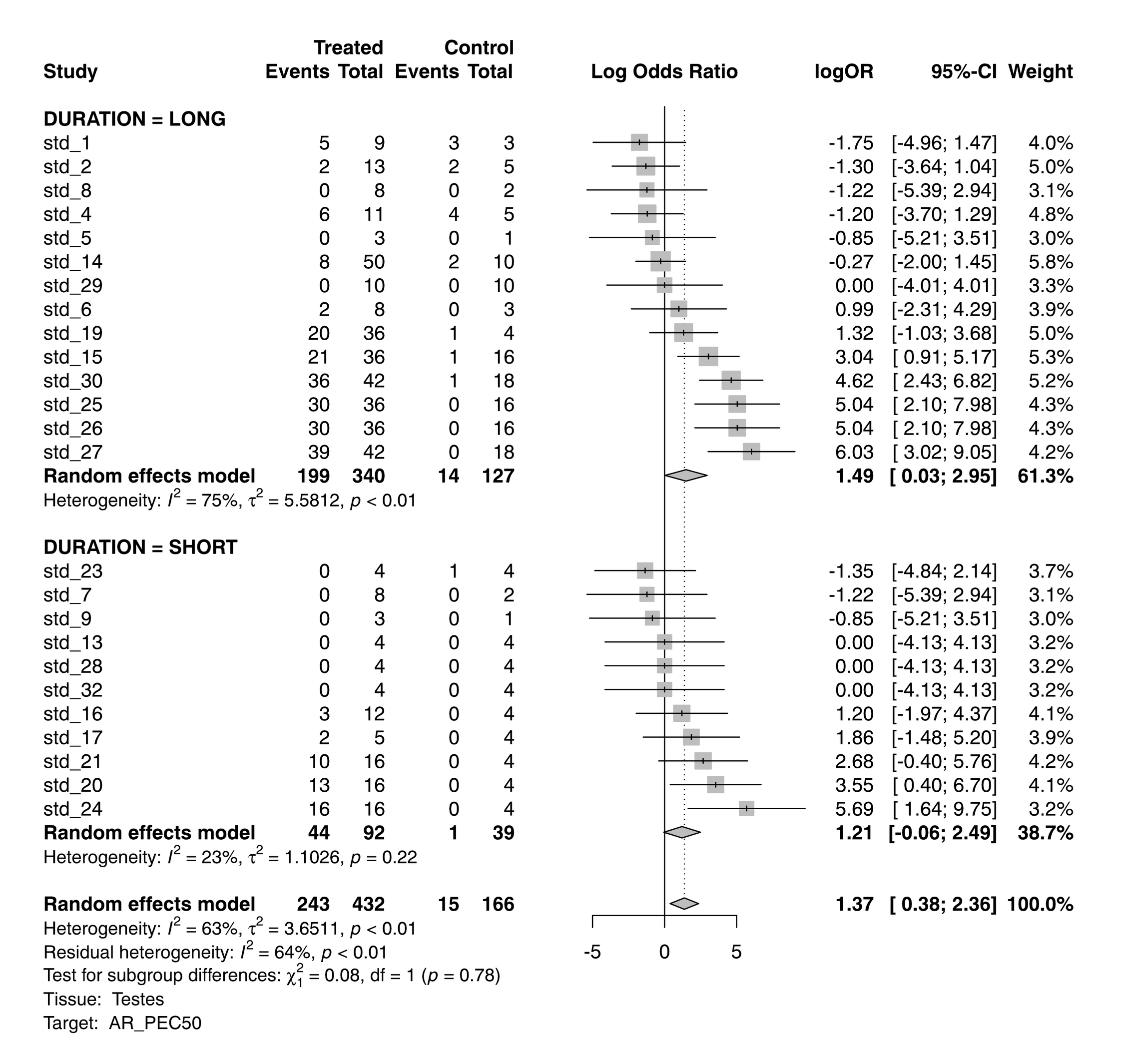

Supplement: S8 Fig — (TIF) [file pone.0252533.s012.tif]

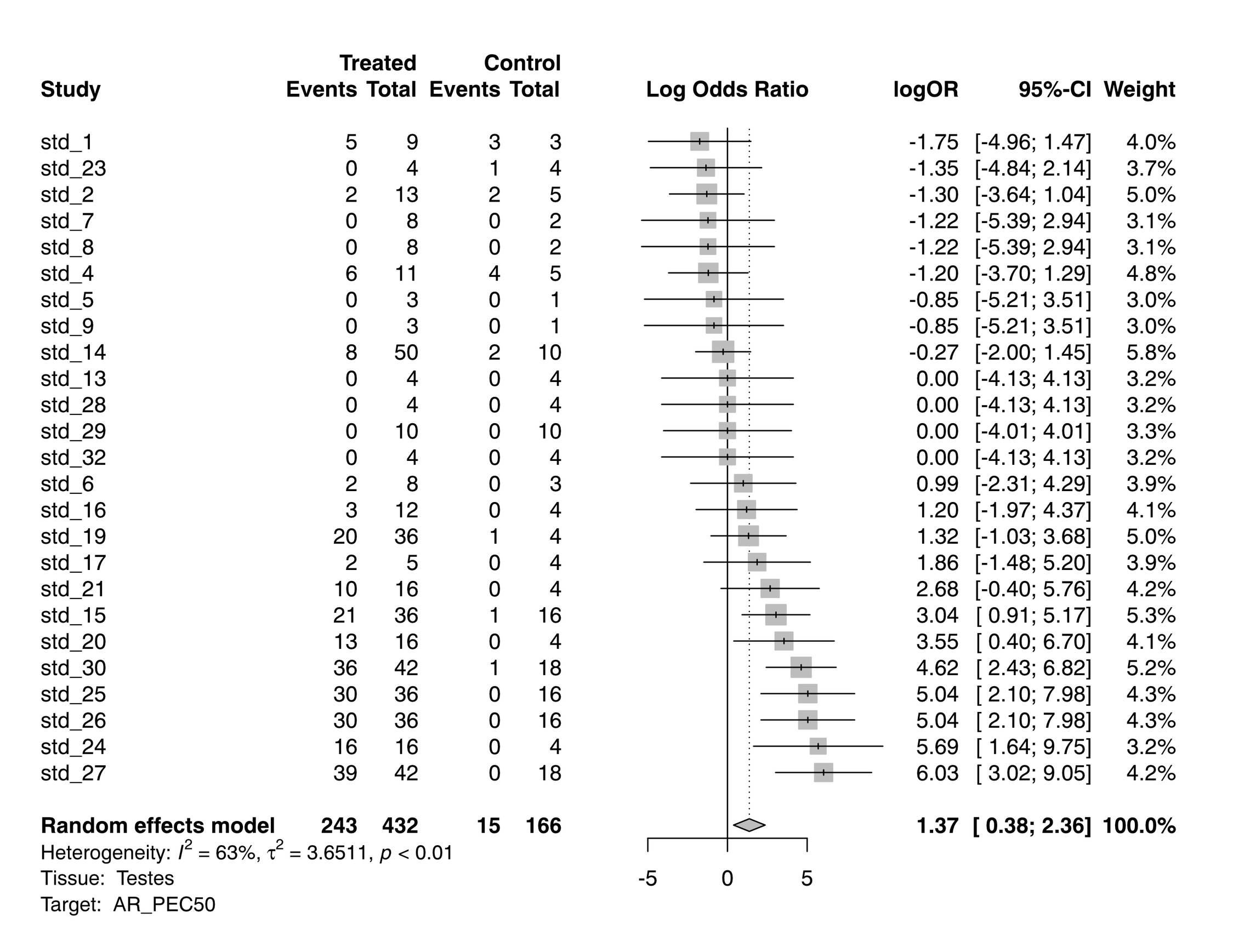

Supplement: S9 Fig — (TIF) [file pone.0252533.s013.tif]

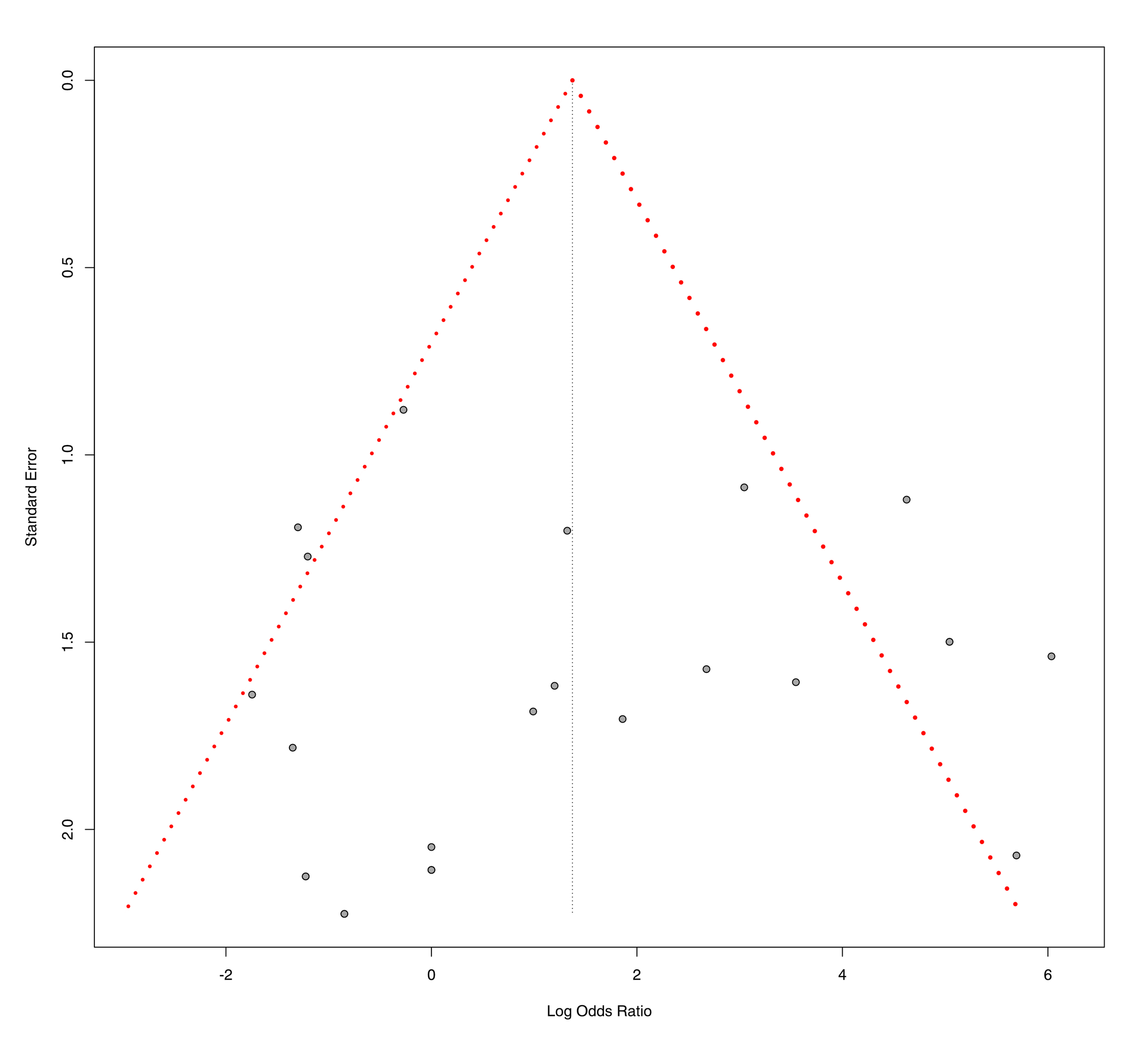

Supplement: S10 Fig — (TIF) [file pone.0252533.s014.tif]

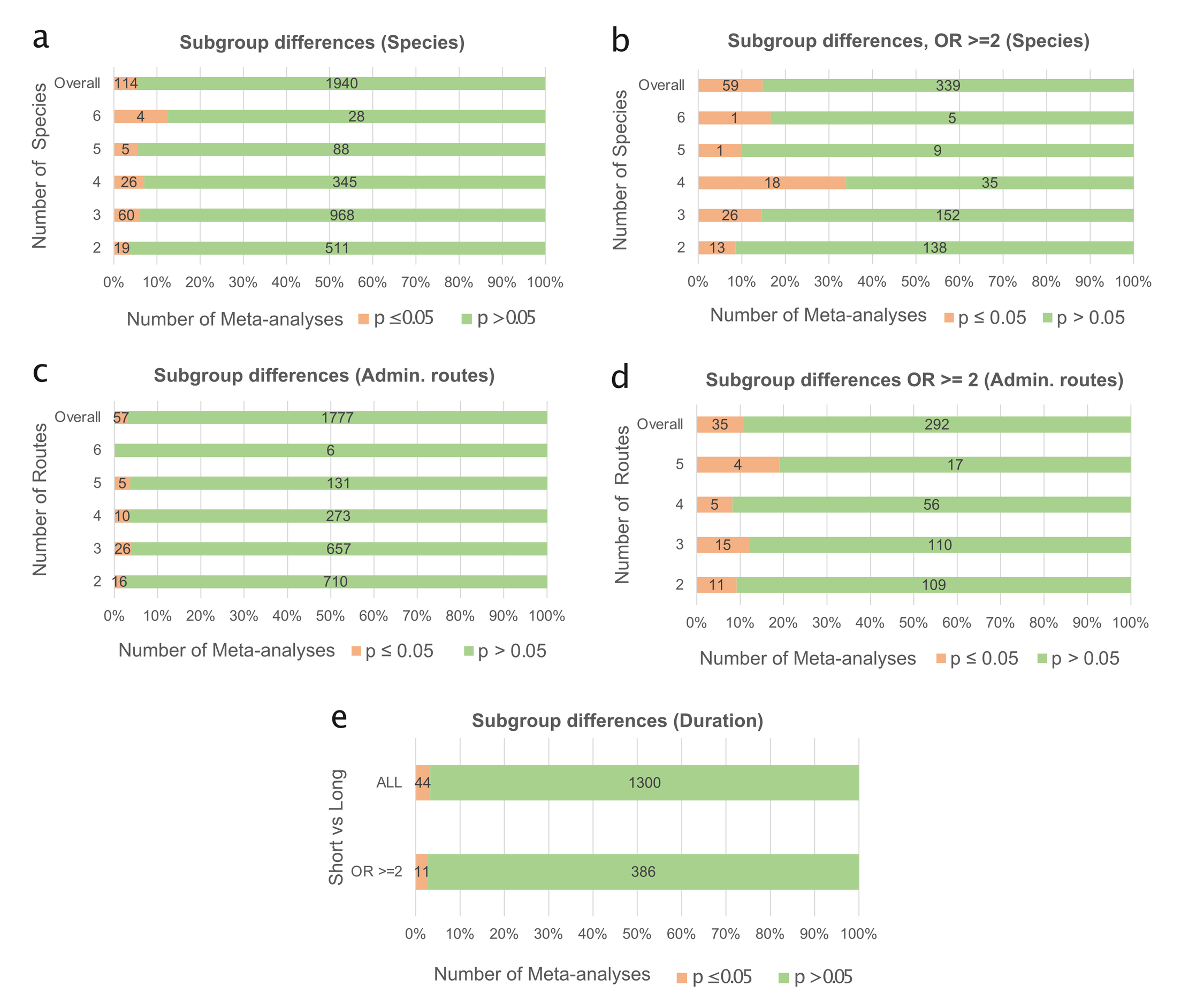

Supplement: S11 Fig — Results are shown for all meta-analysis and also, separately, for meta-analysis with larger effect size (OR > = 2). Results are also disclosed per number of subgroups in on each meta-analysis. (TIF) [file pone.0252533.s015.tif]
