# Supplementary material for: Large scale meta-analysis of preclinical toxicity data for target characterisation and hypotheses generation
Source: PLoS One. 2021 Jun 8;16(6):e0252533. doi: 10.1371/journal.pone.0252533 (PMC8186779; doi:10.1371/journal.pone.0252533)
Supplement: Attachment — Submitted filename: Response_to_Reviewers.docx [file pone.0252533.s017.docx]

**PONE-D-20-39813
Large scale meta-analysis of preclinical toxicity data for target characterisation and hypotheses generation
PLOS ONE**

Dear Dr. Tushar Kanti Dutta

Thank you very much for your kind letter and my apologies for the time it has taken me to come back to you with a reply. I really hope this message finds you well.

I’d like to thank you and the reviewer for the time and interest dedicated to my manuscript and your helpful comments. I’m glad that you are finding the research worth of merit and I hope I’ve been able to address the points raised in your letter to your satisfaction and that of the reviewer.

For the sake of clarity, I decided to include the original comments raised and answer each one there using colour blue. Please find them enclosed with this letter.

Along with this rebuttal letter, please find also included with the submission a file with the manuscript, a marked-up copy highlighting changes (although for your clarification, I must indicate the changes are limited to a few typos, as all comments were pointed at figures and workflows that were distributed as separate files). In addition, all the figures in the manuscript have been remastered from scratch to comply with the resolution and format requirements.

Also, as requested, please find enclosed with my replies my updated Funding and Competing Interests Statements.

Again, many thanks for your interest and time and I’m at your disposal for any question you may have.

Looking forward to hearing back from you.

Yours sincerely,

Jordi Munoz-Muriedas, PhD

Computational Toxicology

Data and Computational Sciences

GlaxoSmithKline

=======================================================

Additional Editor Comments:

Kindly improve upon the quality of figures as suggested by the reviewer.

[JMM] All figures have been remastered from scratch to comply with the resolution and format requirements

Journal Requirements:

1. Please ensure that your manuscript meets PLOS ONE's style requirements, including those for file naming. The PLOS ONE style templates can be found at

https://journals.plos.org/plosone/s/file?id=wjVg/PLOSOne_formatting_sample_main_body.pdf and

[https://journals.plos.org/plosone/s/file?id=ba62/PLOSOne_formatting_sample_title_authors_affiliations.pdf](about:blank)

 [JMM] Styles have been checked

2. Thank you for providing the following Funding Statement:

"JMM is an employee of GlaxoSmithKline. Apart from my salary, I have received no specific funding for this work."

We note that one or more of the authors is affiliated with the funding organization, indicating the funder may have had some role in the design, data collection, analysis or preparation of your manuscript for publication; in other words, the funder played an indirect role through the participation of the co-authors.

If the funding organization did not play a role in the study design, data collection and analysis, decision to publish, or preparation of the manuscript and only provided financial support in the form of authors' salaries and/or research materials, please review your statements relating to the author contributions, and ensure you have specifically and accurately indicated the role(s) that these authors had in your study in the Author Contributions section of the online submission form. Please make any necessary amendments directly within this section of the online submission form.  Please also update your Funding Statement to include the following statement: “The funder provided support in the form of salaries for authors [insert relevant initials], but did not have any additional role in the study design, data collection and analysis, decision to publish, or preparation of the manuscript. The specific roles of these authors are articulated in the ‘author contributions’ section.”

If the funding organization did have an additional role, please state and explain that role within your Funding Statement.

**[JMM] Please find enclosed my updated Funding Statement:** Jordi Munoz-Muriedas conceived and designed the analysis, retrieved the data, performed the calculations, prepared conclusions and wrote the manuscript. The funder provided support in the form of salaries for Jordi Munoz-Muriedas but did not have any additional role in the study design, data collection and analysis, decision to publish, or preparation of the manuscript.

Please also provide an updated Competing Interests Statement declaring this commercial affiliation along with any other relevant declarations relating to employment, consultancy, patents, products in development, or marketed products, etc.

**[JMM]Please find enclosed my updated Competing Interests Statement:** Jordi Munoz-Muriedas is a full-time employee of GlaxoSmithKline. Jordi Munoz-Muriedas has no other competing interests in relation to other companies, organisations or persons. GlaxoSmithKline is a global healthcare company with a portfolio of medicines in respiratory, HIV, immune-inflammatory and oncology therapeutic areas in addition to vaccines and healthcare products.

Within your Competing Interests Statement, please confirm that this commercial affiliation does not alter your adherence to all PLOS ONE policies on sharing data and materials by including the following statement: "This does not alter our adherence to  PLOS ONE policies on sharing data and materials.” (as detailed online in our guide for authors [http://journals.plos.org/plosone/s/competing-interests](about:blank)). If this adherence statement is not accurate and  there are restrictions on sharing of data and/or materials, please state these. Please note that we cannot proceed with consideration of your article until this information has been declared.

[JMM] I can confirm my commercial affiliation does not alter my adherence to all PLOS ONE policies on sharing data and materials.

Please know it is PLOS ONE policy for corresponding authors to declare, on behalf of all authors, all potential competing interests for the purposes of transparency. PLOS defines a competing interest as anything that interferes with, or could reasonably be perceived as interfering with, the full and objective presentation, peer review, editorial decision-making, or publication of research or non-research articles submitted to one of the journals. Competing interests can be financial or non-financial, professional, or personal. Competing interests can arise in relationship to an organization or another person. Please follow this link to our website for more details on competing interests: <http://journals.plos.org/plosone/s/competing-interests>

 [JMM] I’m the sole author of the paper, only my declared competing interests apply.

3) We note that you have indicated that data from this study are available upon request. PLOS only allows data to be available upon request if there are legal or ethical restrictions on sharing data publicly. For information on unacceptable data access restrictions, please see http://journals.plos.org/plosone/s/data-availability#loc-unacceptable-data-access-restrictions.

[JMM] I believe there may have been a misunderstanding as the submission included raw data and code to allow readers to follow and reproduce the analyses as supporting information and it is my understanding the reviewer found it as per answer to question 3. I can see this misunderstanding may arise from the submission questionnaire where the option “No” implies data is only available upon request. The publication provides raw data and code to allow readers to reproduce the methodology presented. I have updated that option in the submission questionnaire.

The manuscript aims to encourage the use of meta-analyses in preclinical environments, especially in large corporate databases, to leverage all the preclinical data available, but also aims to show how this workflow may be useful in precompetitive environments to combine data from different organisations, and, in that sense, hopefully, encourage more organisations to publicly share preclinical data. It would not be feasible for an organisation to share the whole of its historical proprietary preclinical databases, but for the purposes of this publication, a subset has been shared with 38 animal studies related with Androgen receptor agonists (the example the manuscript goes into more detail). This data allows readers to reproduce all the calculations presented (code with all parameters is also provided as supporting information), and, also very important, it potentially allows them to combine the data with their own preclinical raw data for the same target and enhance the meta-analysis if they wish, or apply the workflow to any other target of their choice.

In your revised cover letter, please address the following prompts:

3a) If there are ethical or legal restrictions on sharing a de-identified data set, please explain them in detail (e.g., data contain potentially identifying or sensitive patient information) and who has imposed them (e.g., an ethics committee). Please also provide contact information for a data access committee, ethics committee, or other institutional body to which data requests may be sent.

[JMM] The manuscript doesn’t contain any human/clinical data that requires de-identification. All datasets provided are shared with no restrictions.

3b) If there are no restrictions, please upload the minimal anonymized data set necessary to replicate your study findings as either Supporting Information files or to a stable, public repository and provide us with the relevant URLs, DOIs, or accession numbers. Please see http://www.bmj.com/content/340/bmj.c181.long for guidelines on how to de-identify and prepare clinical data for publication. For a list of acceptable repositories, please see <http://journals.plos.org/plosone/s/data-availability#loc-recommended-repositories>.

[JMM] All relevant data are within the manuscript and its Supporting Information files. A subset of the GSK preclinical database involving the results covered with more detail in the manuscript (results of preclinical studies involving Androgen Receptor agonists) is made available as supporting information. This dataset includes the results of 38 preclinical studies, annotated by species, administration route and duration, with the counts of animals with events observed in control and treated groups by tissue analysed. The data provided allows to reproduce the meta-analyses for the relations involving androgen receptors and provides an idea of the utility of the methodology the paper advocates for. The meta-analyses can be reproduced with the R code also provided.

We will update your Data Availability statement on your behalf to reflect the information you provide.

 [JMM] Thanks

Please review your reference list to ensure that it is complete and correct. If you have cited papers that have been retracted, please include the rationale for doing so in the manuscript text, or remove these references and replace them with relevant current references. Any changes to the reference list should be mentioned in the rebuttal letter that accompanies your revised manuscript. If you need to cite a retracted article, indicate the article’s retracted status in the References list and also include a citation and full reference for the retraction notice.

[JMM] Bibliography has been checked and to the best of my knowledge is complete and correct. No changes made

[Note: HTML markup is below. Please do not edit.]

Reviewers' comments:

Reviewer's Responses to Questions

**Comments to the Author**

1. Is the manuscript technically sound, and do the data support the conclusions?

The manuscript must describe a technically sound piece of scientific research with data that supports the conclusions. Experiments must have been conducted rigorously, with appropriate controls, replication, and sample sizes. The conclusions must be drawn appropriately based on the data presented.

Reviewer #1: Yes

2. Has the statistical analysis been performed appropriately and rigorously?

Reviewer #1: Yes

3. Have the authors made all data underlying the findings in their manuscript fully available?

The [PLOS Data policy](http://www.plosone.org/static/policies.action#sharing) requires authors to make all data underlying the findings described in their manuscript fully available without restriction, with rare exception (please refer to the Data Availability Statement in the manuscript PDF file). The data should be provided as part of the manuscript or its supporting information, or deposited to a public repository. For example, in addition to summary statistics, the data points behind means, medians and variance measures should be available. If there are restrictions on publicly sharing data—e.g. participant privacy or use of data from a third party—those must be specified.

Reviewer #1: Yes

4. Is the manuscript presented in an intelligible fashion and written in standard English?

PLOS ONE does not copyedit accepted manuscripts, so the language in submitted articles must be clear, correct, and unambiguous. Any typographical or grammatical errors should be corrected at revision, so please note any specific errors here.

Reviewer #1: Yes

5. Review Comments to the Author

Please use the space provided to explain your answers to the questions above. You may also include additional comments for the author, including concerns about dual publication, research ethics, or publication ethics. (Please upload your review as an attachment if it exceeds 20,000 characters)

Reviewer #1: The figures presented were not upto the mark in terms of resolution. The meta-analysis workflow may be more lucidly explained so as to appeal to more versatile spectrum of readers who wish to apply similar techniques in their own fields.

[JMM] All the figures have been remastered from scratch to increase resolution and comply with the Journal requirements. All figures have been passed through PACE and processed files are provided.

[JMM] The meta-analysis workflow (Fig 1) has been improved using colours to help readers to identify what parts of it where processes, inputs/outputs, and databases. In addition, a few additional components have been added to the workflow to help readers to understand the shape of the data that will be merged and processed during the analysis, and also to indicate points where scientists interact with the process to specify parameters, like for example inclusion criteria or thresholds for activity. I’m particularly grateful for that comment as has certainly helped to improve the figure and will surely have a positive impact.

6. PLOS authors have the option to publish the peer review history of their article ([what does this mean?](https://journals.plos.org/plosone/s/editorial-and-peer-review-process#loc-peer-review-history)). If published, this will include your full peer review and any attached files.


If you choose “no”, your identity will remain anonymous but your review may still be made public.


**Do you want your identity to be public for this peer review?** For information about this choice, including consent withdrawal, please see our [Privacy Policy](https://www.plos.org/privacy-policy).

Reviewer #1: No

[NOTE: If reviewer comments were submitted as an attachment file, they will be attached to this email and accessible via the submission site. Please log into your account, locate the manuscript record, and check for the action link "View Attachments". If this link does not appear, there are no attachment files.]

While revising your submission, please upload your figure files to the Preflight Analysis and Conversion Engine (PACE) digital diagnostic tool, <https://pacev2.apexcovantage.com/>. PACE helps ensure that figures meet PLOS requirements. To use PACE, you must first register as a user. Registration is free. Then, login and navigate to the UPLOAD tab, where you will find detailed instructions on how to use the tool. If you encounter any issues or have any questions when using PACE, please email PLOS at [figures@plos.org](mailto:figures@plos.org). Please note that Supporting Information files do not need this step.

[JMM] Thanks for the advice. Figures have been processed with PACE. I really found the PACE tool very convenient.
